# Design of Novel Letrozole Analogues Targeting Aromatase for Breast Cancer: Molecular Docking, Molecular Dynamics, and Theoretical Studies on Gold Nanoparticles

**DOI:** 10.3390/metabo13050583

**Published:** 2023-04-23

**Authors:** Alaa Edris, Mohammed Abdelrahman, Wadah Osman, Asmaa E. Sherif, Ahmed Ashour, Elrashied A. E. Garelnabi, Sabrin R. M. Ibrahim, Rawan Bafail, Waad A. Samman, Kholoud F. Ghazawi, Gamal A. Mohamed, Abdulrahim A. Alzain

**Affiliations:** 1 Department of Pharmaceutical Chemistry, Faculty of Pharmacy, University of Gezira, Wad Madani 21111, Sudan; 2 Department of Pharmaceutics, Faculty of Pharmacy, University of Gezira, Wad Madani 21111, Sudan; 3 Department of Pharmacognosy, Faculty of Pharmacy, Prince Sattam Bin Abdulaziz University, Al-kharj 11942, Saudi Arabia; 4 Department of Pharmacognosy, Faculty of Pharmacy, University of Khartoum, Al-Qasr Ave, Khartoum 11111, Sudan; 5 Department of Pharmacognosy, Faculty of Pharmacy, Mansoura University, Mansoura 35516, Egypt; 6 Department of Pharmaceutical Chemistry, Faculty of Pharmacy, University of Khartoum, Al-Qasr Ave, Khartoum 11111, Sudan; 7 Preparatory Year Program, Department of Chemistry, Batterjee Medical College, Jeddah 21442, Saudi Arabia; 8 Department of Pharmacognosy, Faculty of Pharmacy, Assiut University, Assiut 71526, Egypt; 9 Department of Pharmaceutics and Pharmaceutical Technology, College of Pharmacy, Taibah University, Medina 42353, Saudi Arabia; 10 Department of Pharmacology and Toxicology, College of Pharmacy, Taibah University, Medina 30078, Saudi Arabia; 11 Clinical Pharmacy Department, College of Pharmacy, Umm Al-Qura University, Makkah 24382, Saudi Arabia; 12 Department of Natural Products and Alternative Medicine, Faculty of Pharmacy, King Abdulaziz University, Jeddah 21589, Saudi Arabia

**Keywords:** cancer, aromatase, letrozole analogues, molecular docking, molecular dynamics, gold nanoparticles, drug discovery, health and wellbeing

## Abstract

The use of aromatase inhibitors is an established therapy for estrogen-dependent breast cancer in postmenopausal women. However, the only commercially available aromatase inhibitor, letrozole, is not highly selective; in addition to aromatase, it has an affinity for binding to desmolase, an enzyme involved in steroidogenesis, which explains the main side effects. Therefore, we designed new compounds based on the structure of letrozole. More than five thousand compounds were constructed based on the letrozole structure. Then, these compounds were screened for their binding ability toward the target protein, aromatase. Quantum docking, Glide docking, and ADME studies showed 14 new molecules with docking scores of ≤−7 kcal/mol, compared to the docking score of −4.109 kcal/mol of the reference, letrozole. Moreover, molecular dynamics (MD) and post-MD MM-GBSA calculations were calculated for the top three compounds, and the results supported in their interaction’s stability. Finally, the density-functional theory (DFT) study applied to the top compound to study the interaction with gold nanoparticles revealed the most stable position for the interaction with the gold nanoparticles. The results of this study confirmed that these newly designed compounds could be useful starting points for lead optimization. Further in vitro and in vivo studies are recommended for these compounds to verify these promising results experimentally.

## 1. Introduction

Cancer is characterized by an uncontrolled growth of abnormal cells that can appear and metastasize in different tissues of the body, and cancer is currently the second leading cause of death after cardiovascular disease [1]. Breast cancer is now the most prevalent cancer detected worldwide and ranks fifth among the leading causes of cancer-related deaths, having surpassed lung cancer as the most commonly diagnosed cancer with 2.3 million new cases (11.7%). This is followed by lung cancer (11.4%), colorectal cancer (10.0%), prostate cancer (7.3%), and gastric cancer (5.6%). Breast cancer is responsible for 1 in 6 of all female cancer-related deaths and is the leading cause in 110 countries worldwide [2].

It is widely accepted that estrogen plays a crucial role in the progression and metastasis of breast cancer. Specifically, in postmenopausal women, the concentration of 17β-estradiol (E2) in breast cancer may be up to ten times higher than in plasma [3]. This may be due to either increased plasma uptake or in situ androgen aromatization. Aromatase, an enzyme involved in the rate-limiting step of estrogen biosynthesis, catalyzes three successive hydroxylation reactions that aromatize C19 androgens to C18 estrogens. Aromatase is part of the cytochrome P450 enzyme superfamily and is a membrane-bound protein located in the endoplasmic reticulum. Human aromatase (CYP19A1) is located on chromosome 15, band q21.2 of the genome [4].

Therefore, inhibiting estrogen synthesis by blocking aromatase is an advantageous therapeutic approach to treating hormonally sensitive breast cancer [5]. Aromatase inhibitors (AIs) are first-line drugs for the treatment of estrogen receptor (ER)-positive breast cancer in postmenopausal women. Third-generation AIs, including exemestane, anastrozole, and letrozole, have been approved by the Food and Drug Administration as a first-line therapy for hormone-sensitive breast cancer in postmenopausal women, as they have been proven to be superior to tamoxifen, a representative of selective estrogen receptor modulators (SERMs) [6].

However, with extensive application of AIs in clinical practice, unexpected problems have emerged, including a lack of response in some patients, resistance to AI treatment, and inhibition of certain CYP450 enzymes [7]. Letrozole, a reversible third-generation aromatase inhibitor, stops the final step of the conversion of androgens to estrogens [8], with inhibition rates of up to 80–90%. Consequently, letrozole reduces the availability of estrogen in various organs and tissues, including the ovaries, breasts, adipose tissue, and musculoskeletal system [9]. While letrozole markedly inhibits the action of estrogen in breast cancer cells, it also causes systemic effects. For instance, estrogens control the metabolism of lipids and lipoproteins, and a decrease in their production may result in a dysregulation of lipid indices [10].

Computer-aided drug design (CADD) can be used at various stages of drug discovery, including hit identification through virtual screening, optimization of affinity and selectivity, and optimization of other pharmaceutical properties while preserving affinity [11]. CADD comprises two main disciplines: drug structural design (molecular docking and molecular dynamics) and ligand-based drug design (3D quantitative structure-activity relationship (3D-QSAR) and molecular similarity) [12]. To determine the optimal binding conformation, evaluation functions, such as descriptor-based, empirical, power, and knowledge-based approaches, are used. The QM-Polarized Ligand Docking protocol is designed to improve partial charges on ligand atoms during Glide docking by replacing them with charges obtained from quantum mechanical calculations on a ligand in the receptor region. This method differs from molecular docking, which predicts the conformation of ligand binding within the receptor binding site [13,14]. Another CADD method is density-functional theory (DFT), which can solve various problems related to atoms, molecules, and solids, such as calculating the ionization potential, examining vibrational spectra, and selecting catalytically active sites. It can also be used to study the electronic structure of biological molecules and the electronic band structure. Gold-based nanoparticle delivery systems are known for their ability to enhance clinical symptoms of cancer and reduce side effects associated with chemotherapy [15]. Nanoparticles, including hyaluronic acid-based nanoparticles loaded with nutraceuticals, such as curcumin or quercetin, have been reported for cancer treatment [16].

In this study, we developed novel selective aromatase inhibitors against breast cancer using a combination of quantum docking, molecular dynamics, and theoretical studies of gold nanoparticles.

## 2. Methods

In silico studies were conducted using Maestro v 12.8 of the Schrödinger suite [17], while DFT calculations were performed using Gaussian09. 

### 2.1. Protein Preparation 

There are 38 aromatase structures in the Protein Data Bank (PDB), with 27 of them belonging to Homo sapiens. The selected entry for docking was PDB ID 5JKV, which has a more complete amino acid sequence and contains the endogenous ligand ASD in the binding site. The 3D structure of the aromatase protein in complex with testosterone (PDB ID: 5jkv) was obtained and prepared using the Schrödinger Protein Preparation Wizard module following standard procedures [18]. This included assigning correct charges, bond orders, and atom types to the protein structure; deleting water molecules beyond 5 Å from the het group; and filling in missing side chains and loops using the Schrödinger Prime Module [19]. The grid box of the protein’s active site around the bound ligand was generated using the Glide Receptor Grid Generation module [20]. Furthermore, aromatase has a molecule of pentaethylene glycol (PEG) in a secondary allosteric binding site, which weakly inhibits aromatase. Although the haem cofactor remained in place, ASD and PEG were eliminated from the protein [21].

### 2.2. Preparation of the Ligand

Letrozole was prepared using the Schrodinger jaguar utility, which generates low-energy 3D structures at a neutral pH while retaining specific chirality and generating low energy-ring conformers. Hydrogens were added or removed to approximate the pKa values [22,23].

### 2.3. Quantum-Polarized Ligand Docking (QPLD) and Design of New Aromatase Inhibitors

Since the protein bound to letrozole did not have a crystal structure, quantum docking was performed using the QM-Polarized ligand docking method [24]. New letrozole analogues were designed using enumeration, and a new R group library was created by the R group-created panel from the enumeration set at the para position of the phenyl group [25]. The co-crystallized ligand was re-docked against the target protein to validate the docking procedure.

### 2.4. Glide Docking 

The newly designed compounds were then screened against the target protein using LigPrep and subjected to standard precision (SP) docking mode, and the top hits were further subjected to the extra precision (XP) precision mode. 

To predict the binding affinity of the designed compounds for the human off-target desmolase, XP glide docking was used to dock the ligands with the protein.

### 2.5. ADME Prediction

The Schrodinger Qikprop was used to predict the ADME properties of the newly designed compounds, including absorption, distribution, metabolism, and excretion [26].

### 2.6. Molecular Dynamics (MD) Simulation and Post-MD Binding Free Energy Calculation

A molecular dynamics (MD) simulation study was conducted to evaluate the stability of the protein–ligand complexes under physiological conditions, such as solvation, temperature, pH, and pressure. The study used the OPLS4 force field in Desmond, a popular academic software for running MD simulations. The top three letrozole-designed analogs were selected as the input and were neutralized with sodium and chloride ions. A simulated triclinic periodic boundary box with a 10 Å extension in all directions was created for each system. The systems were subjected to energy minimization using the NPT ensemble class, with a gradient threshold of 25 kcal/mol/Å at 300 K and 1 bar pressure. The MD simulation was run for 100 ns under the NPT ensemble class. Long-range coulombic interactions were determined using PME, while the RESPA integrator was used to regulate all covalent bonds connected with hydrogen atoms. A cut-off value of 9.0 Å was selected for short-range electrostatic interactions, and a uniform density approximation was used to analyze long-range van der Waals (VDW) interactions. The Nosé–Hoover thermostat was applied to maintain a temperature of 300 K and 1 atmosphere pressure during the simulation, while the Martyna–Tobias–Klein barostat was used to maintain the conditions. The complexes of the test compounds were compared to the reference ligand by calculating root mean square deviation (RMSD), root mean square fluctuation (RMSF), radius of gyration (Rg), H-bond occupancies, and secondary structure elements (SSE).

After conducting the MD simulation, the Gbind of the docked Combine1, Combine2, and Combine3 complexes were determined using the molecular mechanics generalized the Born surface area (MM-GBSA) module in the Schrodinger suite to evaluate their binding stability. The OPLS4 force field and VSGB solvation model were utilized to compute the binding free energy [27,28]. During the MM-GBSA calculations, a frame was selected at every 10 ns interval after the MD run. The binding free energy was determined using the following equation:

∆G_bind_ = G_complex_ − (G_protein_ + G_ligand_)

where ∆G_bind_ = binding free energy, G_complex_ = free energy of the complex, G_protein_ = free energy of the target protein, and G_ligand_ = free energy of the ligand. 

### 2.7. Preparation of Gold Nanoparticles and DFT Calculations

The study utilized models of (Au) metal clusters as a surface for Combine1 to form a nanostructure. First, the gold cluster and Combine1 were optimized to achieve low energy use. The optimized models were combined to maximize drug relaxation near Au, and the loaded Combine1 with Au was obtained for further research. In addition to optimal geometries and interacting distances, other factors, such as binding affinity, dipole moment, orbital energies, and energy gaps, were investigated for the analyzed models. The DFT computations were carried out using the Gaussian program with Gaussview [29], utilizing the B3LYP/LANL2DZ basis set. Although the following equation was used to calculate interaction energy, other factors were also considered:

Interaction energy = complex energy − gold energy − compound energy

## 3. Results

### 3.1. Molecular Docking and ADME Studies

Molecular docking determines the binding affinity of a compound toward the binding cavity of its receptor. First, the RMSD value of re-docking of the co-crystalized ligand with aromatase (ID: 5jkv) was 0.1193 Å, indicating the accuracy and efficiency of the docking methodology used in this research. 

**Figure 1:**
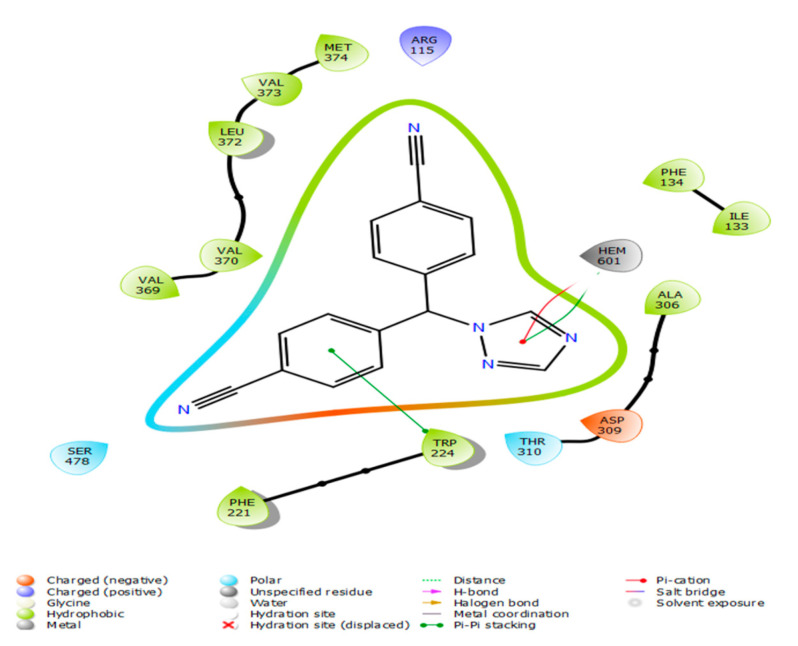
Two-dimensional interaction of letrozole in the active site of aromatase protein (PDB ID: 5jkw) using the QPLD tool of the Maestro software.

In this study, our aim was to find novel letrozole analogues that could serve as inhibitors of aromatase enzymes using in silico studies. Quantum docking of letrozole with the aromatase protein (Figure 1) resulted in the best pose, with a docking score of −4.109 kcal/mol. 

**Figure 2:**
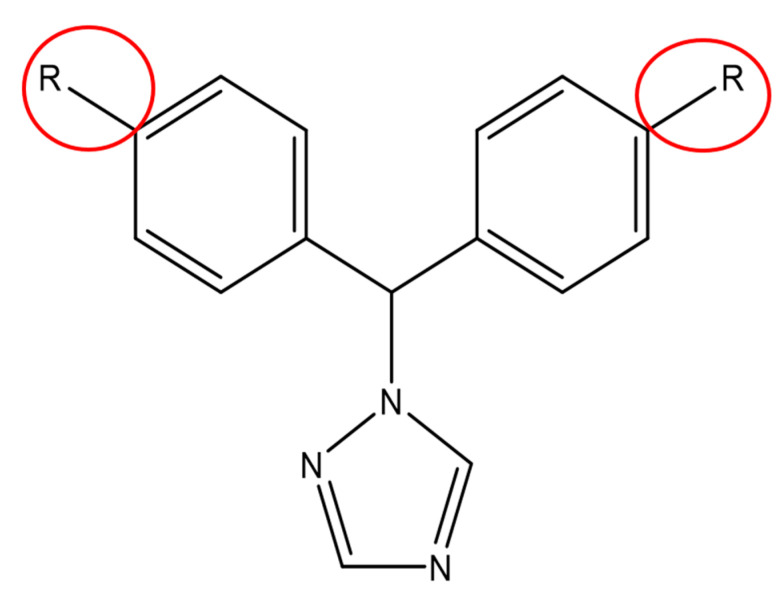
Substitution positions in letrozole based on the R group enumeration.

Based on this pose, we designed new letrozole analogues using diverse R group enumeration (Figure 2), which resulted in 5000 compounds. These compounds were prepared with the LigPrep tool, resulting in 11,204 compounds. 

**Figure 3:**
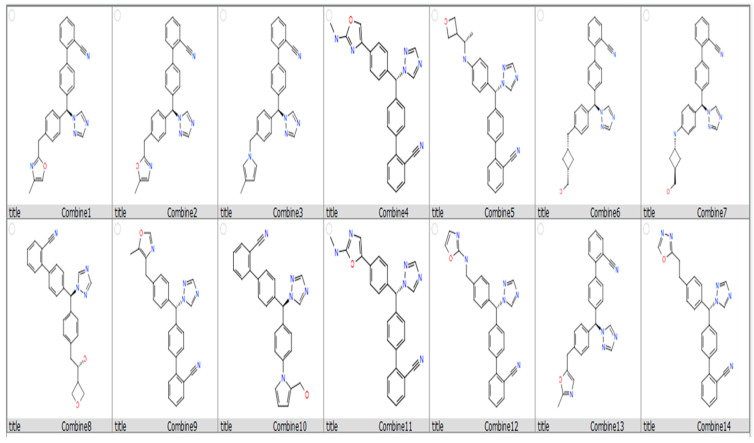
Chemical structures of the 14 analogues that show promising binding affinity against aromatase protein (PDB ID: 5jkw) using the enumeration tool of the Maestro software.

The Glide tool was used to predict the strength, affinity, and molecular interactions of the prepared compounds toward the binding site residues. The prepared compounds were first docked to aromatase using the HTVS mode. Then, the top 500 docked poses with docking scores above −6.00 kcal/mol were further subjected to the SP mode. Finally, the top 200 compounds with docking scores above −7.00 kcal/mol were docked utilizing the XP mode. Table 1 and Figure 3 present the compounds (Combine1–14) with docking scores of −7.041 to −8.117 kcal/mol greater than that of the bound ligand testosterone (−5.322 kcal/mol).

Combine1–14 were further analyzed for their ADME properties, and the Lipinski’s rule was evaluated according to the restrictive rules. According to the Lipinski’s rule of 5, MW ≤ 500, MLogP ≤ 4.15, N or O ≤ 10, and NH or OH ≤ 5 [30]. The Schrodinger’s Qikprop module was utilized to assess the drug-likeness (Lipinski’s rule of five) and ADME evaluations of the designed compounds that exhibited the best binding affinity. Combine1 to Combine14 displayed in Table 2 do not violate the Lipinski’s rule of five. Furthermore, the ADME properties of the compounds were studied; QPlogpo/w ranges from 4.117 to 5.797, and QPPCaco ranges from 313.812 to 946.340. The range of cellular membrane access is −0.952 to −1.741, while the range of QPlogPMDCK is 141.355 to 466.079. The % human oral absorption of these compounds is 100%.

**Figure 4:**
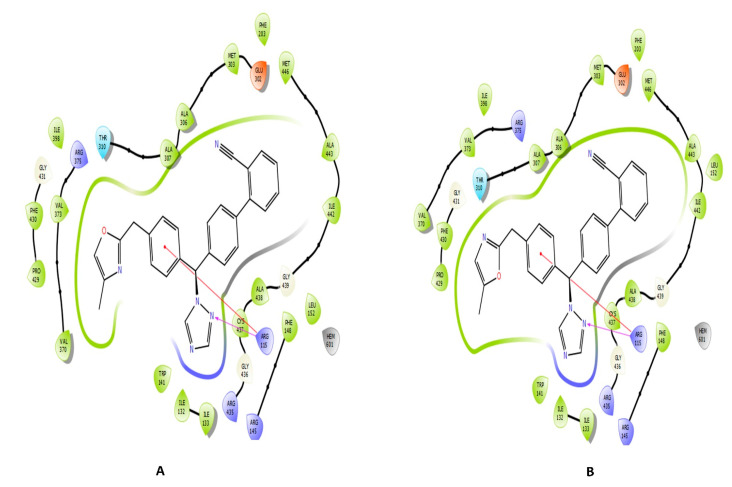

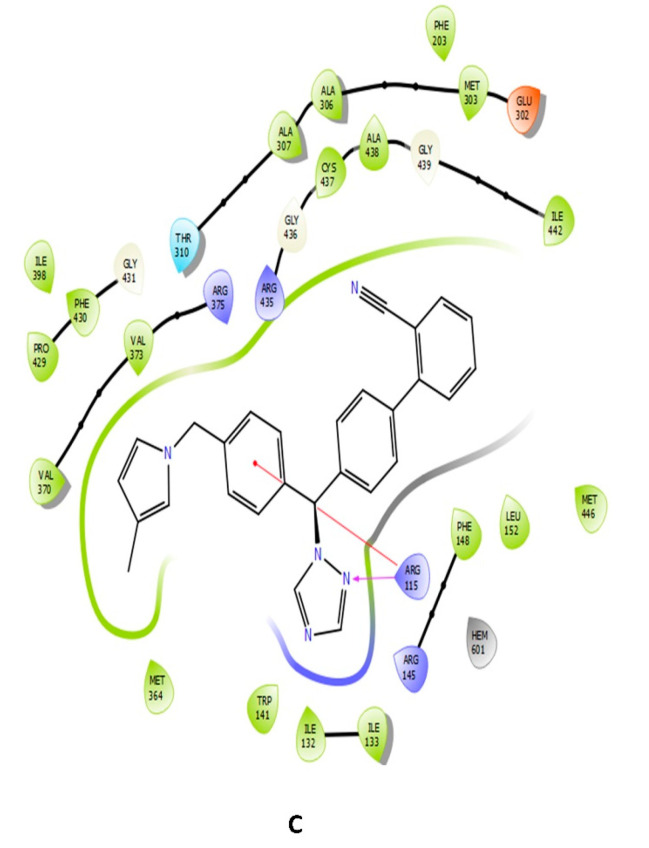
Two-dimensional interactions of the top three analogues with aromatase protein (PDB ID: 5jkw) using the XP docking mode of the Glide software: (**A**) Combine1, (**B**) Combine2, and (**C**) Combine3.

The top three compounds (Combine1–3) with the highest docking scores of −8.117, −8.053, and −8.83 kcal/mol, respectively, and favorable ADME properties (QPlogpo/w, QPlogS, and QPPCaco) were shortlisted for further analysis (Figure 4). These three compounds exhibit a similar interaction pattern with ARG115 through hydrogen bonds and p-cation interactions.

### 3.2. Molecular Dynamics and Post-MD MM-GBSA Calculations

MD simulation was conducted on the compounds that demonstrated stronger ligand–protein interactions, namely Combine1, Combine2, and Combine3, following XP-docking and with the best ADME study results. The changes in structural conformation were monitored in terms of RMSD and RMSF, while the protein interactions with the ligands were monitored throughout the simulation.

**Figure 5:**
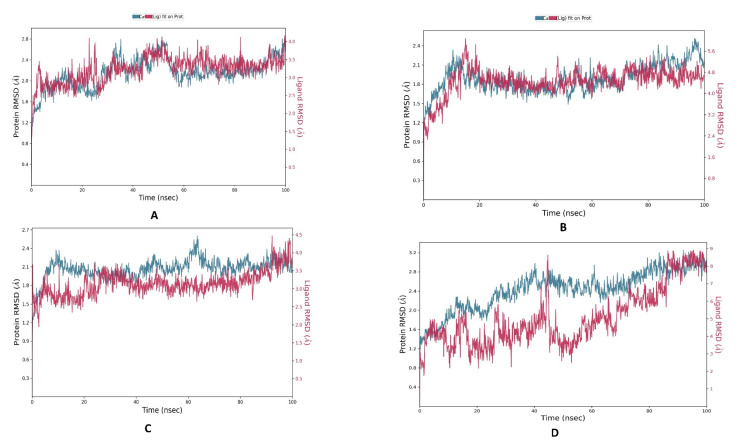
The protein–ligand RMSD plot of the top compound and the reference complexed with aromatase protein (PDB ID: 5jkw) during 100 ns molecular dynamics simulation using the Desmond software: (**A**) Combine1, (**B**) Combine2, (**C**) Combine3, and (**D**) letrozole.

Combine3 has the most fluctuating RMSD curves between 8 and 20 ns, with an RMSD range between 1.94 and 4.17 Å. The RMSD analysis (Figure 5) shows that Combine1 and Combine2 are the most stable among the three complexes, with a medium fluctuation observed. During the entire simulation, the Combine1 complex has an RMSD range between 1.49 and 4.2 Å, and Combine2 has an RMSD range between 1.7 and 6.07 Å. However, the RMSD curves for Combine1 and Combine2 show tight binding with aromatase during most of the simulations, indicating that the conformations obtained from the MD simulations are structurally stable and ideal for further computational analysis when compared to the RMSD of letrozole (0.6–3.1 Å).

**Figure 6:**
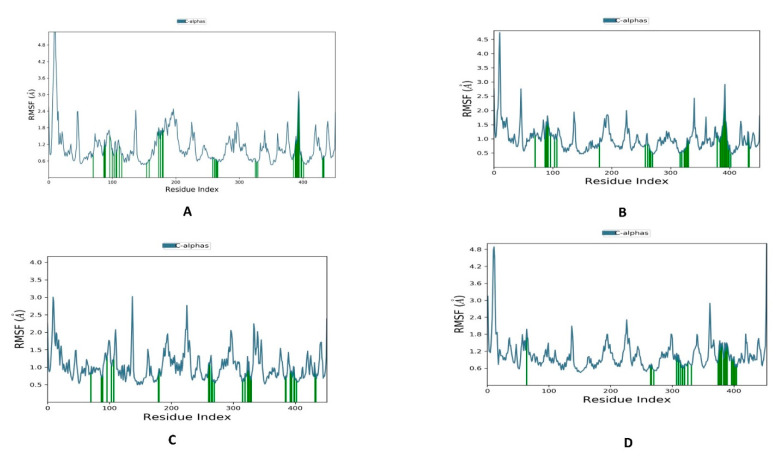
The RMSF plot of the aromatase protein (PDB ID: 5jkw) bound to the top compound and the reference during 100 ns molecular dynamics simulation using the Desmond software: (**A**) Combine1, (**B**) Combine2, (**C**) Combine3, and (**D**) letrozole.

Figure 6 shows that the protein bound to letrozole and the top three compounds has less fluctuation, with values below 2.5 Å. The regions of striking values of RMSF are distant from the binding pocket; hence, it could be inferred that the active site can accommodate the bound compounds without a negative influence on the stability of the binding.

**Figure 7:**
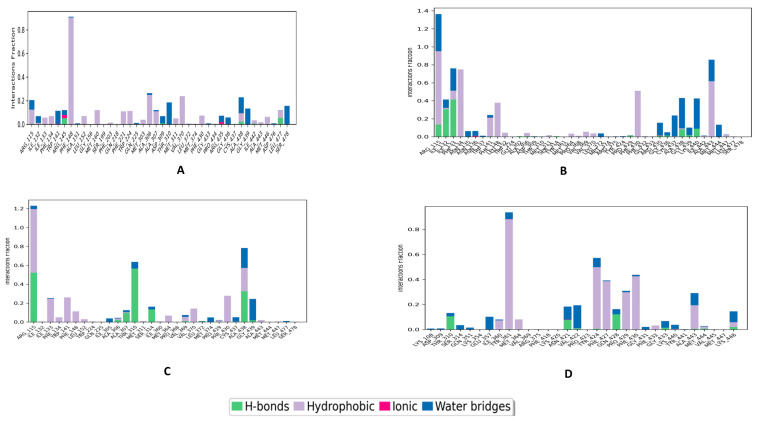
The protein–ligand contacts of the top compound and the reference complexed with aromatase protein (PDB ID: 5jkw) during 100 ns molecular dynamics simulation using the Desmond software: (**A**) Combine1, (**B**) Combine2, (**C**) Combine3, and (**D**) letrozole.

As shown in Figure 7, Combine1 has hydrophobic interactions with PHE148 (90.6%), ALA306 (24.9%), and VAL370 (23.9%). Water bridges are also formed with many residues, such as THR310 (18.4%), ALA438 (13.5%), GLY439 (13.3%), and SER478 (15.8%). Combine2 shows hydrogen bond interactions with ARG115 (13.1%), ILE123 (30.6%), and ILE133 (41.4%). Additionally, it forms hydrophobic interactions with ARG115 (81.5%), PHE134 (74.9%), PHE4330 (50.8%), and PHE148 (37.9%). Combine3 protein complex forms hydrogen bond with ARG115 (52.1%), THR310 (56.6%), and ALA438 (32.6%). Additionally, it shows interactions through water bridges with ALA438 (21.1%) and GLY439 (22.7%). Many hydrophobic interactions are formed with ARG115 (67.3%), PHE430 (27.9%), ILE133 (24.7%), TRP141 (26.1%), and ALA438 (24.8%).

The low RMSD, RMSF, and simulation duration imply that the aromatase protein’s 3D structural model is accurate, and the three complexes are structurally stable and equilibrated. These top three compounds were further subjected to MM-GBSA. MM/GBSA calculates the ligand strain energy by placing the ligand in a solvent, thus giving more reliable results than those calculated from MD results. The post-MD MM-GBSA results for the three compounds and letrozole were calculated from the MD results to be in the range from −68.9075 to −63.5514 kcal/mol (Table 3).

### 3.3. DFT Calculations

The DFT calculations were carried out on Combine1, which has the best MD results. This compound contains five nitrogen atoms and one oxygen atom. The gold model interacts with each of the six atoms, resulting in six complexes (with the exception of Complex1, which contains oxygen), as shown in Table 4. Among these complexes, Complex3, which contains nitrogen, exhibits the strongest binding energy compared to the other complexes.

**Figure 8:**
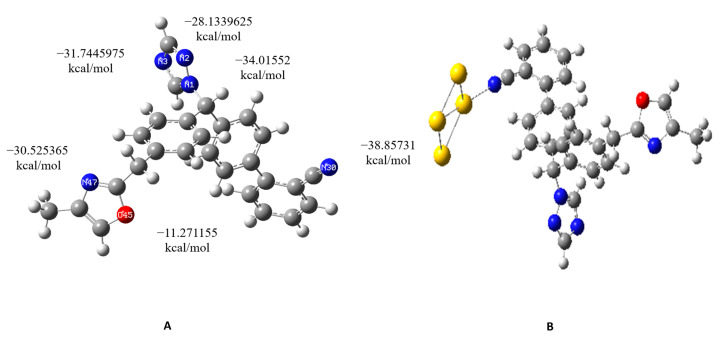
(**A**) Summary of the different interaction energy values of gold atom with the heteroatoms of Combine1. (**B**) Interaction of nitrogen30 with gold cluster.

According to the results shown in Table 4 and Figure 8, Complex3 of Combine1, with a nitrogen atom interacting with the gold cluster, exhibits a binding energy of E(UB3LYP) = −1932.209405, a zero-point energy of −1931.780390, and an interaction energy of −0.061924 hartree (−38.85731 kcal/mol).

### 3.4. Molecular Docking with Human Off-Target Desmolase

Extra-precision Glide docking was employed to dock the proposed designed compound with the human off-target desmolase and predict the binding affinity of the top three ligands for the protein. This resulted in a docking score of −5.3 to −5.4 kcal/mol.

## 4. Discussion 

In 2020, breast cancer was the most frequently diagnosed cancer in women, with an estimated 2.3 million new cases, making it the most incident and deadly malignancy [2]. Estrogen receptor (ER), which is often directly implicated in cancer development, provides the basis for two types of anti-hormone therapy: selective estrogen receptor modulators (SERMs), such as tamoxifen, and selective estrogen receptor degraders (SERDs), such as fulvestrant [31]. The current methodology for estrogen hardening involves limiting aromatase (CYP19A1) to reduce estrogen production, e.g., using letrozole [31,32]. To date, considerable effort has gone into designing novel compounds with advances in IC50 values and selectivity over clinically approved reference compounds, showing promising AI activity and selectivity.

In this investigation, we chose a protease, aromatase, as our target of interest to design new compounds that bind to it with optimal efficacy as a novel treatment for breast cancer. We retrieved the three-dimensional structure of aromatase from the PDB and prepared it for the docking study. We then applied quantum-polarized ligand docking with letrozole, which resulted in a docking score of –4.109 kcal/mol. Based on this, we designed over five thousand compounds based on the letrozole structure and docked them using the Glide tool to study their potential to inhibit aromatase. After the HTVS and SP Glide docking modes, the top hits with docking scores < −7.00 were further subjected to XP docking to confirm their affinity to aromatase. The docking scores of Combine1 to Combine14 show the highest docking scores compared to the reference drug letrozole, as shown in Table 1. The top three compounds (Combine1–3) have docking scores ranging from −8.117 to −8.83 kcal/mol. Combine1–3 interact with ARG115 through hydrogen bonds and pi-cation interactions. Moreover, Combine1 forms hydrophobic interactions with VAL370, VAL373, ALA306, and ALA307; Combine2 forms hydrophobic interactions with ALA306 and ALA307; and Combine3 displays hydrophobic interactions with VAL370 and VAL373. Several reports agreed with these findings, including a study by Naravut Suvannang et al. that demonstrated the docking of aromatases showed hydrophobic interactions between the protein and the AIs involving VAL370, VAL373, ALA306, ALA307, THR310, and MET311, and hydrogen bond interaction involving ARG115 residue [33]. Chanamon Chamduang et al. synthesized and performed in silico studies on a set of 13 unique triazole-tetrahydroisoquinoline derivatives. According to their in silico analysis, the aromatase enzyme’s highly potential inhibitory action might depend on the creation of hydrogen bonds between 2i and THR310 [34]. The same study revealed that the docking results of triazole 1 against aromatase displayed crucial interactions, including hydrophobic interaction and pi–pi stacking interaction using triazole. One of the compounds formed hydrophobic interaction with residue Val370.

Optimizing ADME properties, in addition to their pharmacological effects, increases the success of drug discovery. All compounds, including Combine1–3, complied with the Lipinski’s rule, and all calculated parameters are presented in the supporting data. The theoretical calculations indicate that the ADME parameters of the compounds are within suitable limits, including absorption and distribution across the body, cell permeability, and cellular membrane access. Moreover, they do not cross the blood–brain barrier. QPlogPo/w, which is essential for estimating the absorption and distribution of drugs throughout the body, ranges from 4.117 to 5.797, and QPPCaco, a cellular permeability factor that influences the metabolic pathway, ranges from 313.812 to 946.340. Therefore, these compounds have the potential to behave as drug-like molecules, whereas the reference compound letrozole demonstrates weak cell permeability and low human oral absorption.

During the 100 ns of MD simulation, all ligand–aromatase complexes were used to estimate the stability of the protein–ligand interaction. Root mean square deviation (RMSD) was used to analyze the structural stability of the macromolecular system throughout the simulation time. The higher the stability of the macromolecular system, the lower the values of the RMSD, indicating fewer deviations from the reference mean distance. Despite the excellent results of the docking investigation, which supported our design reasoning, MD studies were carried out for additional confirmation and validation of the entire work. We performed four dynamic simulations to identify and study the nature of aromatase dynamics and compared it with letrozole to provide insights for future lead optimization. Root mean square fluctuations (RMSF) were conducted on the protein complexed with the reference ligand and the three top compounds to measure the stability of amino acid residues. RMSF is a statistical analytical tool that conveys the magnitude of residue motion throughout a simulation, allowing us to understand which regions of the protein are responsible for high amounts of fluctuations. Therefore, the RMSF of the protein main chain was plotted on the *y*-axis against the number of protein residues on the *x*-axis. The minimal changes in the RMSF values suggest that the protein–ligand complexes are structurally stable. The low RMSD values obtained during the whole simulation in the RMSD interpretation of the MD simulations suggest stability for both the protein and its inhibitor complex. The MD simulations produced structurally stable conformations that are suitable for further computational study. These findings were additionally confirmed by determining the free binding energy using the post-MD MM-GBSA method. The MM-GBSA calculations infer that our selected compounds have the most favorable energy with the active site of aromatase, even better than letrozole.

Gold-based nanoparticle delivery systems are known for their ability to amplify clinical symptoms of cancer and reduce the side effects associated with chemotherapy administration [15,35]. In this study, DFT simulations were used to investigate drug loading on metal clusters. Firstly, each of the Combine1 and Au molecular models were optimized to obtain the lowest energy structures. Then, to achieve the most stable configurations of the interacting components, the complex formation of Au and Combine1 was studied by conducting additional optimizations. The outcomes of this study include the obtained values of energies for molecular binding processes, as well as energies of molecular orbital levels and dipole moments. The DFT study showed that Combine1 exhibits stable interactions, as shown in Table 4 where Complex3 (Figure 8) with a nitrogen atom interacting with the gold cluster in position 30 has an interaction energy of −0.061924 hartree (−38.85731 kcal/mol). These results indicate that placing gold atoms at various positions around the heteroatom leads to low-energy complexes, which can be considered as stable. These theoretically stable complexes can guide further testing of experimental gold nanoparticle carriers for Combine1. The gold atom forms a non-covalent bond through interaction with the Combine1 heteroatom. This preliminary non-covalent interaction releases the drug from the engineered nanoparticle carrier once it reaches the active site in an in vivo environment. Further delivery systems, such nano-emulsion stabilized with lecithin and loaded with cobalt ferrite oxide nanocubes, are recommended for cancer treatment [36]. Additionally, targeting other targets related to cancer, such as HER2, with nanoparticles represents a promising approach [37].

## 5. Conclusions 

The aim of this research study was to design novel letrozole analogues with a high affinity for the enzyme aromatase and a lower affinity toward desmolase in order to reduce the undesirable side effects associated with letrozole. To achieve this, 5000 compounds were designed through enumeration, and Glide docking and ADME analyses were used to filter out 14 compounds (Combine1 to Combine14) that showed greater binding affinity than the reference letrozole toward the aromatase protein. Further analysis revealed that Combine1, Combine2, and Combine3 had better molecular dynamics (MD) results compared to the reference letrozole. In addition, the DFT quantum mechanical calculation of the interaction energy between Combine1 and a representative gold atom resulted in stable interaction energy, indicating that these compounds could potentially be formulated as gold nanoparticles. These in silico results suggest that these hits could be used as starting points for lead optimization.

## Figures and Tables

**Table 1:** Docking scores and interactions of the top 14 compounds, letrozole, and testosterone with aromatase (PDB ID: 5jkw).

Name	Docking Scores (Kcal/mol)	Pi-Cation	Hydrogen Bond	Hydrophobic Interaction
Combine1	−8.117	ARG115	ARG115	ALA306/ALA307/MET303/VAL370/VAL373/ARG375/THR310/GLU302
Combine2	−8.053	ARG115	ARG115	PRO429/PHE430/ALA307/ALA306/ALA443
Combine3	−8.83	ARG115	ARG115	ARG435/MET364/ARG375/ARG435/VAL370/VAL373
Combine4	−7.737	ARG115	GLN248	ALA443/MET446/MET311/ILE442
Combine5	−7.704	ARG115	ARG115/PHE430	GLU302/TRP141/MET303
Combine6	−7.411	ARG115	ARG115	VAL373/GLU431
Combine7	−7.342	HEM601	ARG115/ARG375/ARG345	ILE398/PRO429/PHE430/PHE432
Combine8	−7.324	HEM601	PHA430/ARG375	PRO429/PHE430/ALA438/CYS437
Combine9	−7.304		ARG115	MET303/ALA306/ALA307/VAL373/PHE430
Combine10	−7.287		ARG145/PHE430	ALA306/ALA307/ALA438/MET303/ILE398
Combine11	−7.119	ARG115	GLN428	ALA438/GLU439/VAL370/VAL373
Combine12	−7.09		TRP141/ARG115	PRO429/GLY431/ILE398
Combine13	−7.059	ARG115	ARG115	ALA307/ALA438/ALA443/CYS437/PRO429/PHE430/GLY431
Combine14	−7.041		TRP141	ALA306/ALA438/MET303/GLY430/GLY493/ARG375
Letrozole	−4.109	ARG115		MET444/TRY441/TRY424/PHE430/PRO329/PHE427
Testosterone	−5.322		MET374	ILE133, PHE134, PHE221, TRP224, ILE305, ALA306, VAL370, LEU372, VAL373, LEU477

**Table 2:** ADME properties for the top 14 compounds and the reference.

Compound	QPlogPo/w a	QPlogS b	QPPCaco c	QPlogBB d	QPPMDCK e	%HOA	RoF g
Combine1	4.680	−6.913	693.833	−1.167	333.246	100	0
Combine2	4.639	−7.150	463.709	−1.395	215.576	100	0
Combine3	5.797	−8.228	846.562	−1.109	413.198	100	1
Combine4	4.444	−7.404	497.569	−1.291	232.640	100	0
Combine5	4.530	−6.276	681.210	−1.161	326.698	100	0
Combine6	4.715	−6.897	349.469	−1.527	158.792	100	0
Combine7	4.421	−7.404	313.812	−1.741	141.355	100	0
Combine8	4.345	−6.140	602.982	−1.321	286.342	100	0
Combine9	4.566	−6.298	946.340	−0.952	466.079	100	0
Combine10	4.763	−7.227	444.186	−1.340	205.783	100	0
Combine11	4.410	−7.335	402.658	−1.369	185.069	100	0
Combine12	4.397	−5.987	817.738	−1.009	398.012	100	0
Combine13	4.630	−7.118	466.185	−1.387	216.821	100	0
Combine14	4.117	−5.968	437.232	−1.383	202.303	100	0
Letrozole	1.459	−3.693	138.864	−1.570	58.559	73.838	0

**Table 3:** Post-MD MM-GBSA for the top three compounds and Letrozole.

Compound	MM-GBSA Free Binding Energy (kcal/mol)
Combine1	−68.9075 ± 5.304
Combine2	−63.5514 ± 4.219
Combine3	−66.2378 ± 5.1799
Letrozole	−44.3891 ± 8.014567

**Table 4:** DFT results of combine1 and gold atoms.

Complex	Interact Atom	E(UB3LYP)	Dipole Moment	Zero Energy
Complex1	Nitrogen47	−1932.195781	7.401879	−1931.767112
Complex2	Oxygen45	−1932.163889	5.534319	−1931.736428
Complex3	Nitrogen30	−1932.209405	14.785182	−1931.780390
Complex4	Nitrogen3	−1932.197730	8.697839	−1931.769055
Complex5	Nitrogen5	−1932.191763	10.960059	−1931.763301
Complex6	Nitrogen1	−1932.201165	6.402050	−1931.772674
Combine1	-	−1390.215008	6.376844	−1389.789215
Gold	-	−541.930470	0.00	−541.929251

## Data Availability

The datasets generated during and/or analyzed during the current study are available from the corresponding author upon reasonable request. The data are not publicly available due to privacy.
